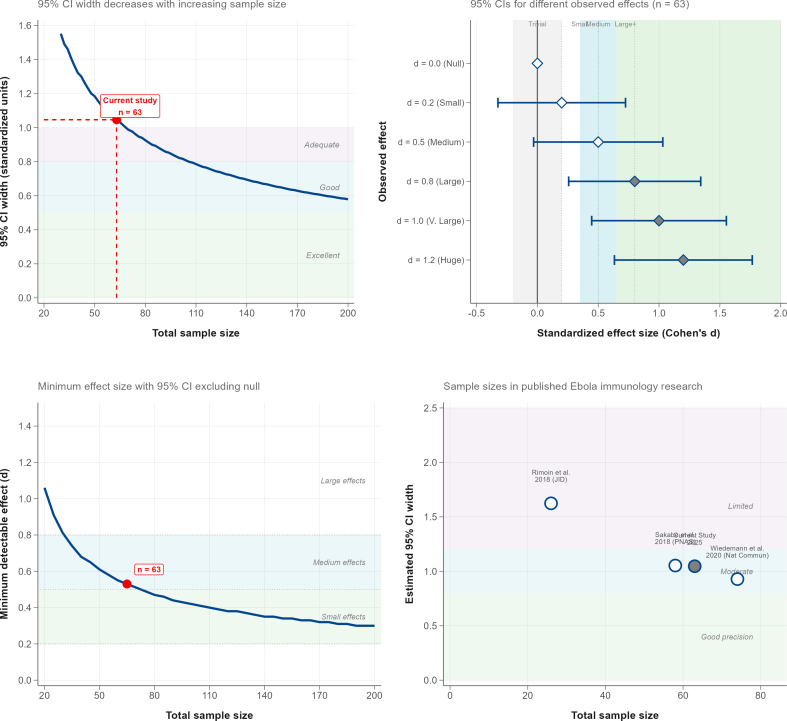# Resilience and residuals beyond containment — The hidden burden of Bundibugyo Ebola virus survivorship sixteen years on: A cross-sectional observational study

**DOI:** 10.1016/j.nmni.2025.101685

**Published:** 2025-12-13

**Authors:** Raymond Ernest Kaweesa, Joseph Ssebwana Katende, Raymond Reuel Wayesu, Annie Daphine Ntabadde, Solomon Opio, Laban Kato, Gerald Kevin Oluka, Ruth Nambi, Rodney Abraham Tumusiime, the FiloStudy Team, Pontiano Kaleebu, Julius Julian Lutwama, Jennifer Serwanga

**Affiliations:** a Department of Immunology, Uganda Virus Research Institute, Entebbe, Uganda; b Uganda Virus Research Institute and London School of Hygiene & Tropical Medicine (MRC/UVRI & LSHTM) Uganda Research Unit, Entebbe, Uganda; c London School of Hygiene and Tropical Medicine, Keppel Street, London, WC1E 7HT, United Kingdom

**Keywords:** Bundibugyo ebolavirus, Ebola disease (EBOD) survivors, Post-ebola syndrome, Long-term viral sequelae, Immunometabolic findings, Survivorship care models, Epidemic preparedness

## Abstract

**Background:**

Long-term effects of Ebola disease (EBOD) are well documented for Ebolavirus (EBOV), but limited data exist for Bundibugyo virus disease (BVD) caused by Bundibugyo Ebola virus (BDBV), a genetically distinct strain.

**Methods:**

We conducted a cross-sectional observational study involving 40 laboratory-confirmed BVD survivors and 23 age- and sex-matched unexposed community controls to evaluate the long-term clinical, biochemical, immunological, and psychosocial sequelae associated with BDBV infection. Participants underwent comprehensive clinical evaluations, laboratory testing, and standardised mental health assessments. Statistical comparisons used rank-sum, chi-squared, and correlation analyses.

**Findings:**

Survivors exhibited persistent multisystem symptoms, with neurological and musculoskeletal complaints most frequent, headaches (35 %) and visual disturbances (22.5 %). Laboratory findings showed elevated basophils (40 %) and urinary ketones (5 %), indicating possible chronic inflammation and metabolic shifts. Respiratory rates were significantly reduced in survivors (p < 0.001), while other vital signs and biochemical markers were largely within normal ranges. Despite high resilience, 70 % with normal anxiety scores and 62.5 % with normal depression scores, 57.5 % reported persistent stigma. Survivors also exhibited unique physiological correlations, suggestive of post-infectious homeostatic changes.

**Conclusion:**

BVD survivors experience long-term multisystem sequelae and physiological remodeling. These findings support the need for virus-specific post-BVD care and sustained follow-up to inform survivor health policy.

## Highlights

• Sixteen years post-infection, Bundibugyo Ebola virus survivors show persistent respiratory and psychosocial symptoms.
• Cardiometabolic and thermoregulatory parameters remain largely within normal ranges.
• Subtle biochemical changes suggest low-grade inflammation and metabolic stress.
• Findings highlight the need for long-term clinical follow-up of filovirus survivors.

## 1 Introduction

Emerging and re-emerging Infectious diseases pose profound challenges to global health, not only during acute outbreaks but also through long-term health consequences that extend well beyond the initial infection. Among these, Ebola disease (EBOD), caused by viruses within the *Filoviridae* family [1,2], remains one of the most devastating, with outbreaks marked by high mortality and severe clinical manifestations. While global health strategies have predominantly focused on the containment and acute management of EBOD outbreaks, growing evidence underscores the profound and long-term health burdens faced by survivors. EBOD survivorship is increasingly recognised as a distinct clinical entity, characterised by persistent physical and psychosocial sequelae that extend far beyond the acute phase of illness.

The long-term health complications associated with Ebola virus disease (EVD) are well-documented for Ebolavirus (EBOV), the most studied variant to date. Survivors of EVD have been reported to experience an array of chronic health issues, including ocular disease [3,4], musculoskeletal pain [5], neurological impairments [6], and mental health challenges such as anxiety, depression [[7], [8], [9]], and post-traumatic stress disorder (PTSD) [9]. These sequelae, collectively referred to as Post-Ebola Syndrome (PES) [10], highlight the persistent impact of viral pathogenesis and immune dysregulation even after viral clearance.

In contrast, survivors of Bundibugyo Ebola virus (BDBV), a lesser-studied strain, remain underrepresented in the literature, despite the severe and enduring health challenges they face. BDBV emerged during Uganda's outbreak from August 2007 to February 2008, marking the first appearance of this genetically distinct Ebola species. Prior characterisation of long-term outcomes following Sudan Ebolavirus (SUDV) and Marburg virus (MARV) infections has demonstrated persistent clinical and immunological sequelae among East African survivors [11,12], yet comparable data for BDBV remain almost entirely absent. Diverging by approximately 32 % in nucleotide sequence from its closest relatives [13,14], BVD exhibited unique clinical and epidemiological characteristics. Like other strains of Ebola, BDBV is highly contagious, primarily spreading through direct contact with the bodily fluids of infected individuals [15] or contaminated objects [16]. The incubation period typically ranges from 2 to 21 days [17], beginning with an nonspecific febrile illness characterized by fatigue, fever, and myalgia. As the infection progresses, patients often develop severe gastrointestinal symptoms including nausea, vomiting, and diarrhea, which can escalate to multi-organ dysfunction [18]. Despite its lower case-fatality rate (34 %) compared to EBOV [19], the chronic sequelae among survivors reveal substantial long-term health burdens, including blurred vision, hearing loss, and joint pain [20], alongside viral persistence in immune-privileged sites such as the eyes [21] and testes [22], raising the risk of recurrence and sexual transmission after recovery [23]. These findings suggest that BDBV, like its counterparts, contributes to a spectrum of chronic health challenges that warrant comprehensive investigation.

Despite the severity of these outcomes, BVD has not been prioritised in global health discussions, and its survivors remain largely excluded from research and intervention strategies that have driven advancements in therapeutics, vaccination, and clinical care for other Ebola strains. This study seeks to address this critical gap by comprehensively examining the clinical and psychosocial sequelae of BVD survivors from the 2007–2008 outbreak in Uganda's Bundibugyo district. Through systematic clinical assessments and qualitative interviews, we aim to characterise the frequency, nature, and lived impact of post-EVD manifestations. This contributes to a more holistic understanding of EBOD as a multisystemic disease with lasting health implications. Additionally, this study aims to inform survivor-centered policies, strengthen health systems, and enhance epidemic preparedness strategies that extend beyond viral containment to address long-term health equity.

## 2 Materials and methods

### 2.1 Study design and population

We conducted a cross-sectional observational study among two cohorts recruited from communities in Uganda affected by the 2007–2008 BVD outbreak. The survivor cohort consisted of 40 individuals with documented recovery from laboratory-confirmed BDBV infection. A comparator group of 23 age- and gender-matched, unexposed, uninfected individuals was enrolled from the same geographical region to control for environmental and socioeconomic confounders. Controls reported no prior exposure to BDBV or any other Ebola virus species. All participants provided written informed consent before study enrollment. Ethical approval was obtained from the Uganda Virus Research Institute Research Ethics Committee (UVRI-REC, GC/127/1045) and the Uganda National Council for Science and Technology (UNCST, HS5212ES). The study was conducted in accordance with the Declaration of Helsinki and the Good Clinical Practice (GCP) guidelines. All samples were de-identified and securely bio-banked for future immunovirological analyses.

### 2.2 Participant Selection and eligibility criteria

Participants were identified from verified Uganda Ministry of Health outbreak records and local public health documentation. Survivors were eligible if they had laboratory-confirmed BDBV infection during the 2007–2008 outbreak, were residing in Bundibugyo or neighbouring districts at the time of this study and provided written informed consent. Individuals unable to consent or who had relocated outside accessible districts were excluded. Tracing and recruitment were conducted in collaboration with local health authorities and representatives of a recognised survivor network, which maintained updated registries of individuals involved in the 2007–2008 outbreak response. A total of 42 survivors were identified; two were confirmed deceased, and 40 were successfully traced and enrolled.

Community members from Bundibugyo District were screened as potential unexposed controls. Eligibility criteria required being aged ≥17 years, currently residing within Bundibugyo District, able to provide informed written consent, and, specifically for controls, having no self-reported symptoms consistent with Ebola virus disease and no known contact with confirmed cases during the 2007–2008 outbreak. Of 30 individuals screened, seven did not meet inclusion criteria (three were aged <17 years, two declined participation, and two had incomplete data), resulting in 23 eligible participants who were enrolled as unexposed controls. This observational study is reported in accordance with the STROBE (Strengthening the Reporting of Observational Studies in Epidemiology) statement for cross-sectional studies. A completed STROBE checklist is provided (**Supplementary Annex 1**), and a STROBE flow diagram appears as Fig. 1.

Serological testing for BDBV-specific IgG was not used as a basis for classifying exposure status. Given that nearly 17 years had elapsed since the outbreak, antibody titres would likely have waned below detection thresholds in many previously infected individuals, limiting assay reliability for distinguishing prior exposure from true naïve status. Classification, therefore, relied on contemporaneous outbreak documentation and verified Ministry of Health case records, which remain the most definitive method for retrospective survivor identification.

### 2.3 Matching of survivors and controls, demographic balance, and sample size considerations

All 40 traceable BVD survivors from the 2007–2008 outbreak registry [24] were enrolled, representing the complete accessible survivor population. Among the 42 originally confirmed cases, two were deceased, and none of the remaining survivors declined participation. For comparison, 30 community members from Bundibugyo District were screened, of whom 23 met eligibility criteria and were enrolled as unexposed, uninfected controls (Fig. 1). Controls were frequency-matched to survivors by age group and sex, but individual matching was constrained by the national population's demographic structure, one of the youngest globally, with a national median age of 16.9 years and approximately 80 % of citizens under 35. Consequently, fewer older adults were eligible for enrollment as controls, leading to demographic differences between groups (survivors: median 48.5 years, 57.5 % male; controls: 38.0 years, 26.1 % male).

**Fig. 1:**
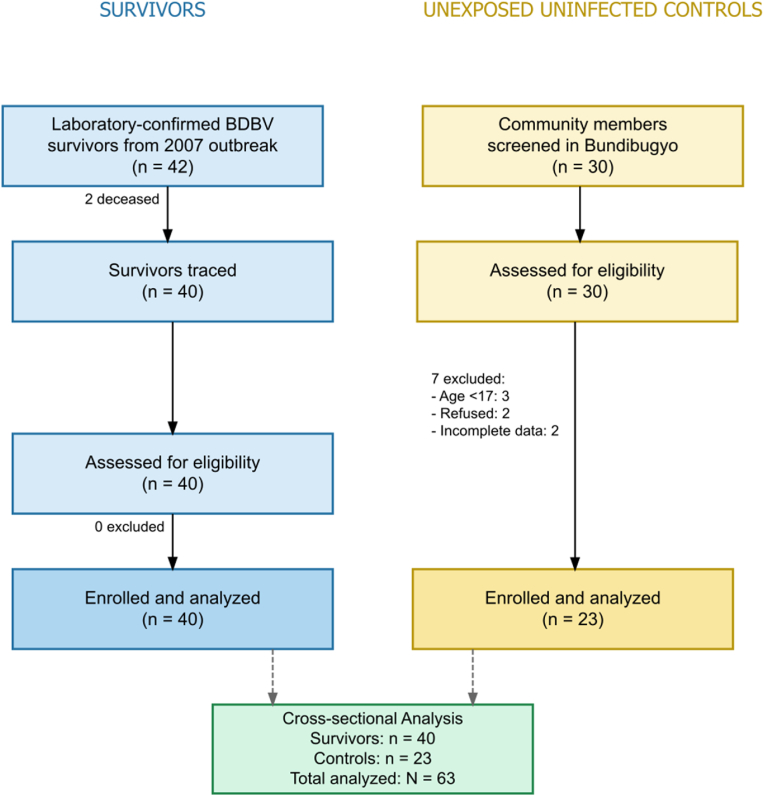
**STROBE Flow Diagram of Participant Selection and Enrollment** This flow diagram illustrates the sequential process of participant identification, screening, eligibility assessment, and enrollment for the Bundibugyo Ebola virus (BDBV) survivor study. The left panel outlines the tracing and enrollment of laboratory-confirmed BDBV survivors from the 2007 outbreak, while the right panel depicts the screening and inclusion of community members serving as unexposed controls. Each stage presents the number of participants screened, excluded, and ultimately included in the cross-sectional analysis. Eligibility criteria, summarised in the upper right corner, include age ≥17 years, residence in Bundibugyo District, ability to provide informed consent, and absence of Ebola virus disease symptoms in 2007 for controls.

To account for these imbalances, all primary analyses were adjusted for age and sex, and partial correlations were computed controlling for these covariates. As shown in Supplementary Fig. S1, adjusted and unadjusted correlations are closely aligned along the line of identity, confirming that the observed associations reflect genuine post-BVD effects rather than demographic confounding. Notably, certain associations, such as mean corpuscular haemoglobin concentration (MCHC), strengthened after adjustment, indicating that underlying survivor effects were not artefacts of age or sex disparities.

Given the rarity of Bundibugyo Ebola virus disease and the small number of surviving individuals, a precision-based approach was employed for sample size justification rather than traditional power calculations [25,26], summarised in Supplementary Fig. S2. With 40 survivors and 23 controls (total n = 63), the study achieved a 95 % confidence interval width of ≈1.05 standardised units for group comparisons, calculated using the variance formula of Hedges and Olkin [27]. This provides adequate precision to detect medium-to-large effects (d ≥ 0.53) and aligns with sample sizes used in comparable Ebola survivor studies [[28], [29], [30], [31]]. Effect sizes are reported as Hedges' *g* with 95 % confidence intervals to facilitate meta-analytic integration [32,33].

### 2.4 Data collection and clinical assessment

Sociodemographic data, including age category, sex, and occupation, were collected using standardised questionnaires. Comprehensive clinical assessments captured long-term symptoms and physiological parameters. Occupations were classified post hoc according to international classification standards. Vital signs, such as heart rate (60–100 bpm), respiratory rate (12–20 breaths/min), temperature (36.1–37.2 °C), BMI (18.5–24.9 kg/m^2^), and blood pressure (systolic 90–120 mmHg, diastolic 60–80 mmHg), were recorded and compared against established reference thresholds [34,35]. Survivors self-reported hallmark sequelae, including fatigue, myalgia, arthralgia, neurocognitive dysfunction, sensory impairments, and gastrointestinal symptoms. Symptom severity (none, mild, moderate, severe) and duration (years) were systematically documented.

### 2.5 Laboratory and immunological evaluation

Venous blood and midstream urine samples were collected under sterile conditions. Hematologic profiles were assessed using Complete Blood Counts (CBC), while renal (creatinine, urea) and hepatic (bilirubin, Alanine Transaminase (ALT), Aspartate Transferase (AST) function tests were performed to evaluate organ health. Inflammatory markers, including C-reactive protein (CRP), were quantified to assess systemic immune activation and residual inflammation. Abnormalities were classified based on internationally accepted thresholds [34,35]. Peripheral blood mononuclear cells (PBMCs) were isolated and cryopreserved for future downstream immunophenotyping of B-cell memory subsets and T-cell activation profiles to elucidate immune remodeling following BDBV infection.

### 2.6 Mental health and social outcomes

Psychological evaluations were performed using the Hospital Anxiety and Depression Scale (HADS), a validated instrument with predefined cutoffs: 0–7 (normal), 8–10 (mild), 11–15 (moderate), and ≥16 (severe) [36]. Binary indicators captured social dimensions, including perceived post-recovery stigma and levels of community or familial support.

### 2.7 Statistical analysis

Descriptive statistics were used to summarise demographic and clinical characteristics. Differences in physiological, biochemical, and psychological outcomes between BVD survivors and age- and sex-matched community controls were assessed using appropriate statistical tests. Continuous variables were analysed with the Wilcoxon rank-sum test, while categorical variables were compared using Pearson's chi-squared or Fisher's exact test. Gender-stratified analyses explored sex-based differences in physiological parameters. Associations between clinical symptoms and laboratory values were evaluated with Spearman's rank correlation. All statistical tests were two-tailed, with significance set at p < 0.05. Data analysis was performed using R version 4.2.0. Missing data were reported per outcome; analyses were conducted using available cases without imputation (HADS missingness: 5 %. Given the census-like inclusion of all traceable survivors and the descriptive aims, no sensitivity analyses were pre-specified; age/sex-adjusted partial correlations and visual inspection of adjusted vs unadjusted effects (Supplementary Fig. S1) served as robustness checks.

## 3 Results

### 3.1 Study population and demographic characteristics

**Fig. 2:**
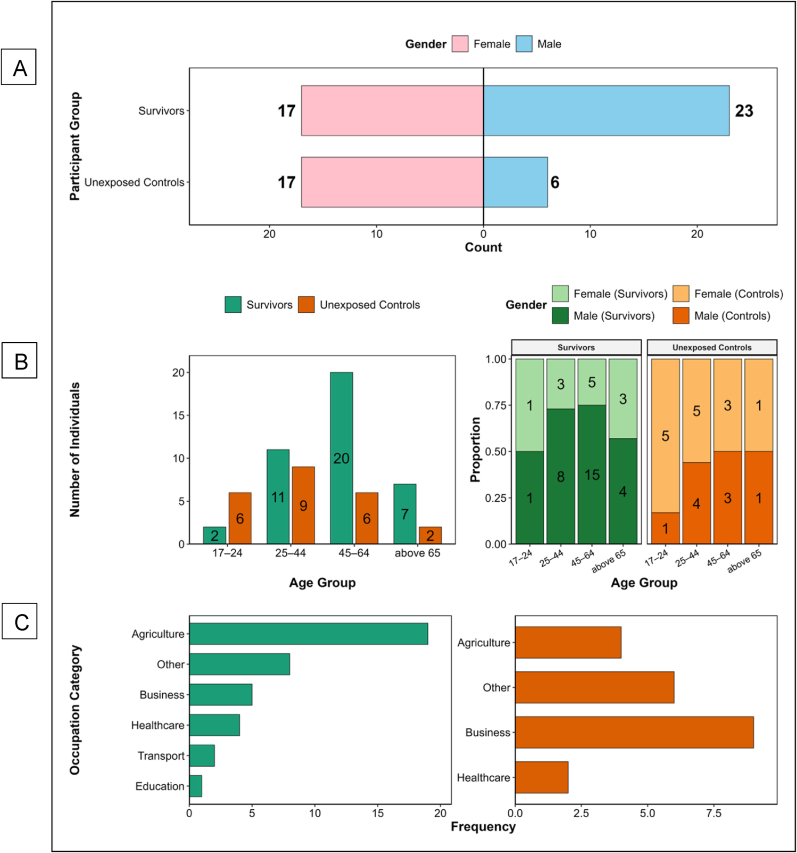
**Sociodemographic Characteristics of Bundibugyo Ebola Virus Survivors and Unexposed Controls** This figure summarises the demographic and occupational profiles of participants enrolled in the cross-sectional study. Fig. 2A displays the gender distribution, showing the number of male (blue) and female (pink) participants within the survivor and unexposed groups. Fig. 2B presents the age across defined brackets (17–24, 25–44, 45–64, and ≥65 years), comparing group composition by both frequency and proportion. Fig. 2C summarises the distribution of occupational categories, highlighting representation across agriculture, business, healthcare, education, transport, and other sectors. Together, these panels describe the demographic structure and socioeconomic diversity of the study cohort.

Between December 4, 2024, and February 10, 2025, we enrolled 63 participants, comprising 40 survivors of laboratory-confirmed BDBV infection and 23 unexposed, seronegative individuals from the same outbreak-affected communities (Fig. 2A). Survivors were defined as individuals with documented recovery from the 2007–2008 Bundibugyo District BDBV outbreak in Uganda. The unexposed group consisted of age- and gender-matched, diverse community members with no known BDBV exposure, serving as an internal demographic and environmental reference population.

Survivors were older than unexposed controls (median age: 48.5 years [IQR 40.0–60.5] vs. 38.0 years [IQR 25.0–49.0]; *p* = 0.008, Wilcoxon rank-sum test). The gender distribution differed between cohorts. Among survivors, males predominated (23/40, 57.5 %), while females represented the majority in the unexposed group (17/23, 73.9 %; *p* = 0.020, Fisher's exact test), Fig. 2B. Education levels did not differ significantly between the groups (*p* = 0.176). However, a notable minority of survivors (15.0 %) reported having no formal education compared to none among controls. The proportion attaining college or university education was higher in the unexposed group (39.1 %) than in survivors (25.0 %).

Self-employment, particularly in agriculture, was the most common employment status among survivors (65.0 %) and controls (56.5 %). Although employment patterns were not significantly different (*p* = 0.572), detailed occupational analysis revealed compelling trends. Agriculture was the dominant occupation among survivors (n = 20), and business-related roles were more prevalent among unexposed controls. Healthcare workers, transport workers, and those in education were underrepresented, but they were disproportionately represented among survivors. Survivors were more likely to be married (77.5 % vs. 59.1 %), while unexposed individuals had a higher proportion of single participants (36.4 % vs. 12.5 %), Fig. 2C. Although this difference did not reach statistical significance (*p* = 0.087), the trend may reflect social support structures influencing care-seeking and survival.

### 3.2 Lower respiratory rates but normal cardiovascular and metabolic indices among ebola bundibugyo survivors

**Fig. 3:**
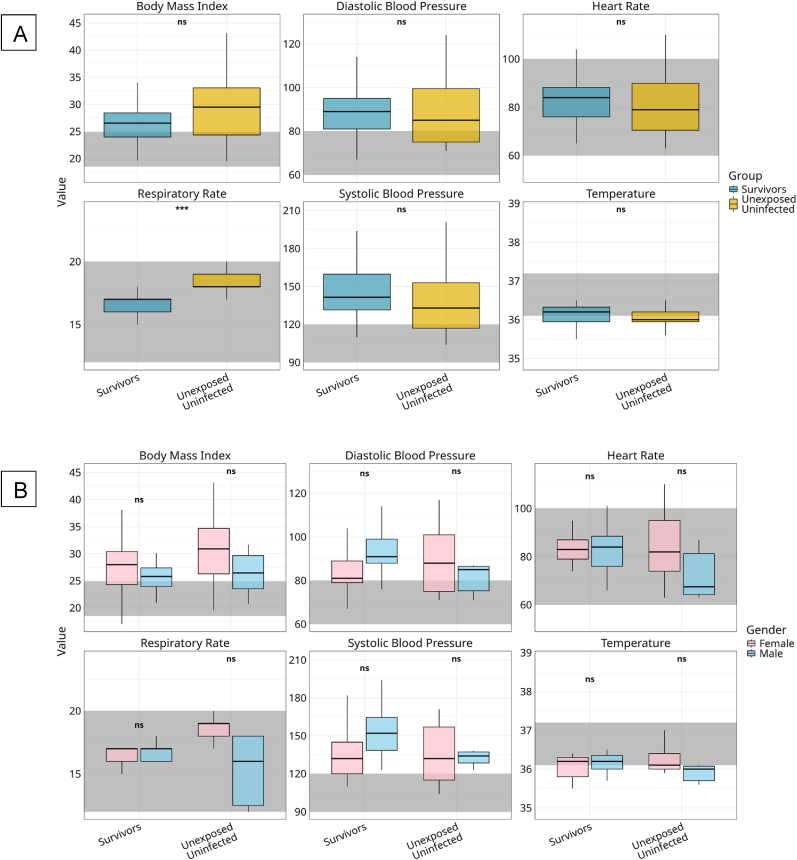
**Vital Signs among BVD Survivors and Unexposed Controls, Stratified by Group and Gender** Fig. 3 compares core physiological parameters between Bundibugyo virus disease (BVD) survivors and unexposed, uninfected controls. Box plots compare body mass index (BMI), systolic and diastolic blood pressure, heart rate, respiratory rate, and temperature between BVD survivors and unexposed, uninfected controls (A). Corresponding distributions are shown stratified by gender within each group (female, pink; male, blue), (B). Boxes represent interquartile ranges with medians; whiskers extend to 1.5 × the IQR, and grey shaded areas denote standard clinical reference ranges. Group and gender comparisons were performed using Kruskal–Wallis tests with Bonferroni correction (*p* < 0.05).

Comparison of vital signs between BVD survivors and matched unexposed controls revealed a statistically significant difference in respiratory rate (p < 0.001), with survivors showing lower rates (Fig. 3A) that remained within the normal physiological range (12–20 breaths per minute). In contrast, several unexposed individuals, particularly females, had elevated rates exceeding this threshold (Fig. 3B). Gender-stratified analysis confirmed significantly higher respiratory rates in unexposed females compared to males (p < 0.05); however, these differences fell within acceptable clinical limits and were not deemed physiologically significant. Other vital parameters did not differ significantly between groups but indicated clinically relevant trends. Body Mass Index (BMI) was consistently higher in unexposed controls than in survivors, with females in both groups showing higher median values than males. Despite these differences, all medians remained within or slightly above the normal range (18.5–24.9 kg/m^2^), suggesting comparable nutritional status across cohorts. Systolic and diastolic blood pressures were marginally higher in survivors compared to unexposed controls, particularly among male participants. Although these differences were not statistically significant, the upward trend in systolic pressure may reflect subtle post-infectious vascular changes.

Heart rate did not differ significantly between groups, however, gender-based patterns emerged. Females across both cohorts exhibited higher median heart rates than males, with the highest values recorded among unexposed females. All measurements remained within normal clinical limits (60–100 beats per minute), indicating no overt autonomic dysregulation. Temperature readings were tightly clustered within the normal physiological range (36.1–37.2 °C) across all groups and genders, suggesting an absence of acute febrile illness at the time of evaluation. Taken together, these data suggest that BVD survivors maintain stable cardiometabolic and autonomic profiles, apart from a significantly reduced respiratory rate.

### 3.3 Long-term symptoms following bundibugyo ebola virus infection showing prevalence, severity, and chronicity among survivors

**Fig. 4:**
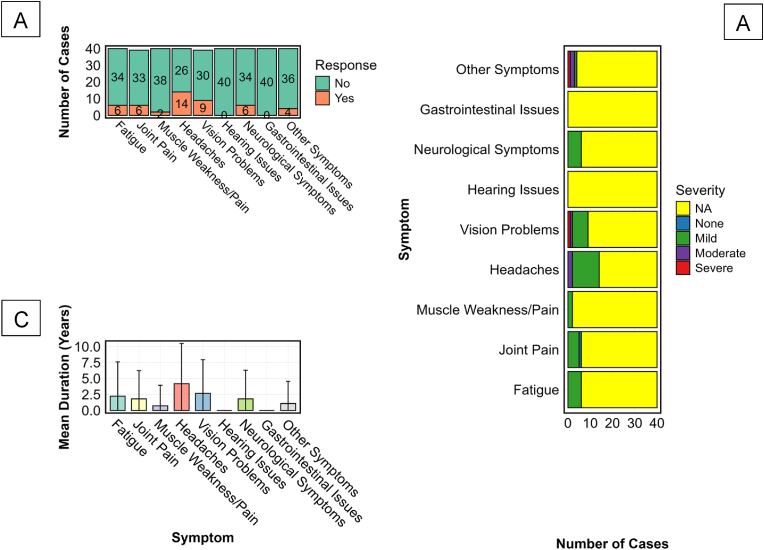
Frequency, Severity, and Duration of Persistent Symptoms Among BVD Survivors NB: One participant who was a baby at the time of the outbreak was not interviewed for symptoms recalling. This three-panel figure summarises the prevalence, severity, and duration of post-EBOD sequelae among 40 BVD survivors. Fig. 4A shows the proportion of participants reporting persistent symptoms, including fatigue, joint pain, muscle weakness, headaches, vision problems, neurological, and other systemic complaints. Fig. 4B illustrates the distribution of symptom severity, classified as mild (green), moderate (purple), or severe (red), where data were available. Fig. 4C presents the mean duration of reported symptoms in years, with error bars indicating standard deviation. Hearing and gastrointestinal symptoms were not reported in this cohort and thus are absent across all panels.

A detailed analysis of persistent post-BVD symptoms in survivors of BDBV infection revealed a clinically significant burden of long-term sequelae with varying patterns of prevalence, severity, and chronicity (Fig. 4). Among 40 confirmed BVD survivors evaluated, post-disease sequelae were common and diverse, with neurological and musculoskeletal symptoms being the most prevalent. Headaches were the most reported symptom, experienced by 35 % (n = 14/40) of survivors, followed by vision problems in 22.5 % (n = 9/40). Fatigue, joint pain, and neurological issues, such as paresthesia or cognitive disturbances, each occurred in 15 % (n = 6/40), while musculoskeletal weakness was noted in 5 % (n = 2/40). Other unspecified symptoms were present in 10 % (n = 4/40). No participants reported hearing loss or gastrointestinal disturbances, suggesting a potential tropism that spares the auditory and enteric systems, Fig. 4A.

Severity assessments, although incomplete, revealed predominantly mild manifestations. Of those reporting headaches, 85.7 % (n = 12/14) described them as mild, and 14.3 % (n = 2/14) as moderate. Vision disturbances were likewise predominantly mild (n = 7/9), with isolated reports of moderate (n = 1) and severe (n = 1) presentations. For most other symptoms, severity data were either unavailable or too limited to categorise conclusively, reflecting a need for more systematic long-term symptom grading in outbreak survivors, Fig. 4B.

Symptom duration data highlight a pattern of protracted morbidity in a subset of survivors. Headaches exhibited the longest mean duration at 4.18 years (SD = 6.30), followed by vision disturbances (2.65 years, SD = 3.20) and joint pain (1.80 years, SD = 2.55). Notably, the wide standard deviations suggest substantial individual variability in recovery trajectories, with some survivors experiencing resolution within months while others endure multi-year impairment, Fig. 4C. Absence of duration data for hearing and gastrointestinal complaints corroborated their non-occurrence in this cohort.

Collectively, these findings illustrate the clinical persistence and heterogeneity of post-BVD syndrome, emphasising the need for tailored long-term care pathways and informing prognostic expectations for survivors. The absence of certain symptom domains may reflect virus-specific tissue tropism, while the persistence of others highlights the need for long-term clinical follow-up and rehabilitation planning.

### 3.4 Persistent but subtle laboratory perturbations in ebola bundibugyo virus survivors

Comprehensive clinical laboratory profiling revealed select physiological distinctions between BVD survivors (n = 40) and unexposed, uninfected controls (n = 23), suggesting long-term immune-metabolic recalibration in a subset of parameters post-recovery. However, most measured values fell within normal ranges in both cohorts, underscoring substantial resolution of acute-phase dysfunction.

**Fig. 5:**
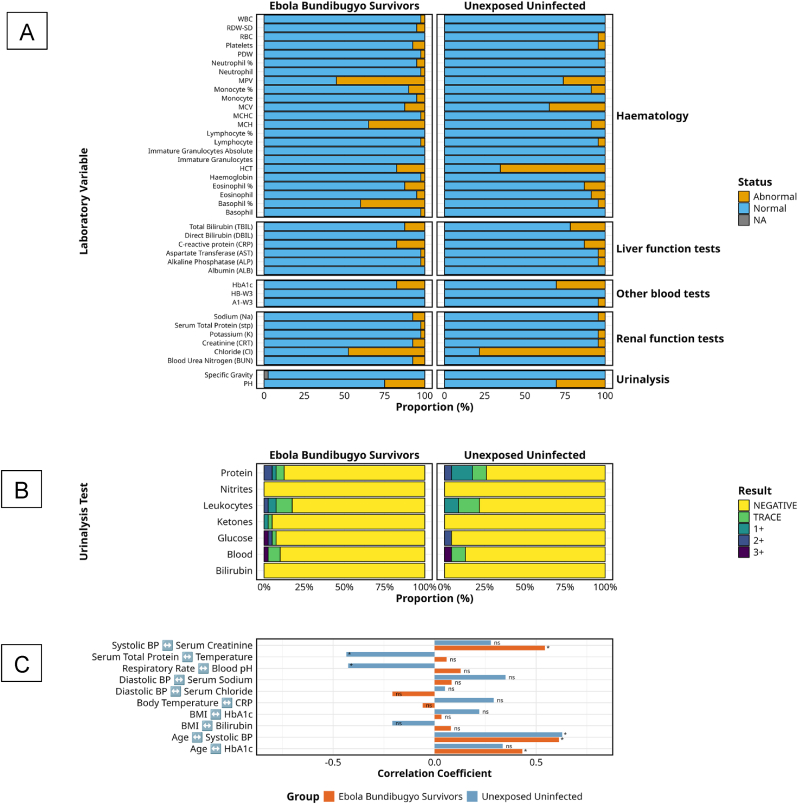
**Clinical Laboratory Profiles and Correlations in BVD Survivors and Unexposed Controls** A summarises the proportion of normal (blue) and abnormal (orange) results across haematology, liver function, renal function, other blood tests and urinalysis among BDBV (n = 40) and unexposed controls (n = 23). Fig. 5B presents detailed urinalysis comparisons for protein, nitrites, leukocytes, ketones, glucose, blood, and bilirubin, graded as negative, trace, or positive (1+ to 3+), indicating trace and positive findings. Fig. 5C shows Spearman correlation coefficients between key laboratory parameters, vital signs and physiological indices, including blood pressure, body mass index, temperature, and HbA1c, illustrating patterns of association within and between study groups.

Haematological recovery was nearly complete (Fig. 5A), parameters were largely comparable between groups, with both survivors and controls showing normal red and white blood cell counts in more than 97.5 % of cases. Haemoglobin abnormalities persisted in only 2.5 % of survivors, indicating near-complete restoration of oxygen-carrying capacity. However, a higher proportion of survivors exhibited elevated basophils (40.0 % vs 4.3 %), potentially indicating persistent low-grade inflammation or altered innate immune tone. Conversely, hematocrit abnormalities were unexpectedly more frequent among the controls (65.2 %) than among the survivors (17.5 %). Monocyte percentages remained largely comparable between groups.

Liver function markers were predominantly within normal limits in both cohorts. Aspartate aminotransferase (AST) and alkaline phosphatase (ALP) were normal in 97.5 % of survivors, mirroring control values. While total bilirubin abnormalities were slightly lower in survivors (12.5 %) compared to controls (21.7 %), this difference was not clinically meaningful. Direct bilirubin and albumin levels were universally normal. C-reactive protein (CRP), although not a conventional liver marker, showed mild elevation in 17.5 % of survivors and 13.0 % of controls, further supporting limited differential systemic inflammation.

Renal function profiles showed only subtle differences (Fig. 5B). Abnormal chloride levels were observed in 50.0 % of survivors and 78.3 % of controls, representing the greatest disparity between groups but not directly attributable to prior BDBV infection. Creatinine and blood urea nitrogen abnormalities were infrequent (7.5 % of survivors, 4.3 % of controls), and electrolyte disturbances (sodium, potassium) were minimal across both groups. Serum total protein levels were consistently normal.

Urinalysis revealed overlapping yet distinctive abnormalities (Fig. 5C). Survivors demonstrated a greater diversity of glucose abnormalities (7.5 % vs 4.3 %) and uniquely exhibited detectable ketones (5 %). Both groups showed comparable rates of hematuria (∼10–13 %) and leukocyturia (17.5 % in survivors vs 21.7 % in controls), with survivors displaying more pronounced leukocyte grading. Proteinuria was paradoxically more prevalent among controls (26.1 % vs 12.5 %), predominantly in the 1+ range. Additional metabolic indices suggested modest survivor-specific deviations. Haemoglobin A1c (HbA1c), indicative of average blood glucose levels, abnormalities were more common in controls (30.4 %) than survivors (17.5 %), raising implications for glucose metabolism potentially unrelated to BDBV exposure.

Correlative physiological analysis revealed lasting changes in survivor physiology. Among survivors, age strongly correlated with systolic blood pressure (r = 0.61, p < 0.001) and HbA1c (r = 0.43, p = 0.005), patterns not seen in controls. Notably, systolic blood pressure in survivors correlated with serum creatinine (r = 0.54, p < 0.001), possibly reflecting long-term renal-vascular interaction. Survivors did not show the significant correlations observed in controls between serum total protein and temperature (r = 0.038, p < 0.05) and between respiratory rate and blood pH (r = 0.043, p < 0.05), suggesting altered homeostatic regulation after infection. No significant associations between BMI and bilirubin or HbA1c were found in either group.

Taken together, these findings indicate that while core organ function has largely normalised, BDBV survivors exhibit discrete immunological, renal, and metabolic differences, particularly in innate cell profiles and physiological integration, suggesting long-term imprinting of host systems following infection. However, given the absence of consistent elevations above control levels, causality to BDBV infection remains cautious.

## 4 Mental health and social integration among long-term ebola bundibugyo survivors

**Fig. 6:**
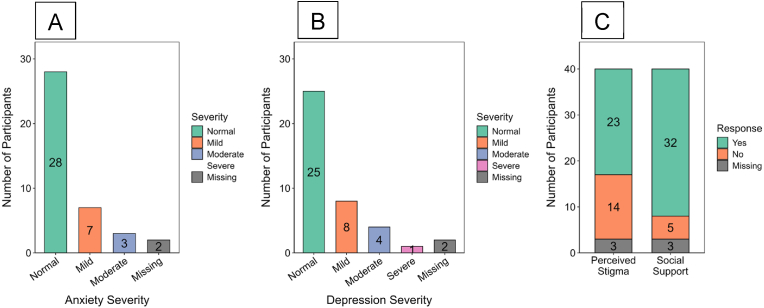
**Mental Health and Psychosocial Profiles Among BVD Survivors** NB: Percentages calculated among respondents with non-missing data. Fig. 6 presents the distribution of anxiety and depression severity, alongside perceived stigma and social support among 40 long-term survivors of BDBV infection in Uganda. The figure includes three panels: Panel A illustrates severity categories of anxiety; Panel B shows depression severity levels; and Panel C depicts self-reported experiences of stigma and access to social support. All measures were obtained through standardised mental health and psychosocial assessment tools. These outcomes reflect individual-level reporting and are not benchmarked against unexposed controls; therefore, attribution to prior BDBV infection cannot be inferred.

Among the 40 BVD survivors assessed approximately 16 years after the outbreak, most individuals displayed mental health resilience across both anxiety and depression domains (Fig. 6). Anxiety severity assessments indicated that 28 (70 %) participants had scores within the normal range, while 7 (17.5 %) reported mild symptoms and 3 (7.5 %) reported moderate symptoms. Two cases (5 %) had incomplete data, Fig. 6A. Depressive symptomatology showed similar trends, with 25 (62.5 %) survivors reporting normal levels. Mild and moderate depressive symptoms were found in 8 (20 %) and 4 (10 %) individuals, respectively, while one survivor (2.5 %) exhibited severe depression. Data were missing for 2 individuals (5 %), Fig. 6B. Social reintegration was marked by perceived psychosocial strain, with 23 (57.5 %) survivors reporting experiences of stigma, while 14 (35 %) reported none. Social support structures were generally preserved, as 32 survivors (80 %) indicated access to supportive relationships, while 5 (12.5 %) did not, Fig. 6C. Three participants (7.5 %) had incomplete responses across psychosocial metrics.

## 5 Discussion

This study presents one of the most comprehensive characterisations to date of the long-term clinical, biochemical, immunological, and psychosocial sequelae in survivors of Bundibugyo Ebola Virus Disease (BDBV) infection, 16 years after the 2007–2008 outbreak in Uganda. The principal finding is that BVD survivorship is marked by persistent but heterogeneous multisystemic sequelae, particularly neurological and musculoskeletal symptoms, alongside subtle immunometabolic perturbations and remarkable mental health resilience. These findings substantiate BVD survivorship as a clinically meaningful post-viral state, deserving of distinct recognition within post-EBOD syndrome (PES) frameworks.

Neurological and musculoskeletal symptoms, primarily chronic headaches (35 %) and vision disturbances (22.5 %), dominated the clinical picture. Although mild in severity, their long-term persistence confirms previous reports of post-EBOD complications from BDBV and EBOV outbreaks [20,37]. Interestingly, no survivors in our cohort reported hearing loss or gastrointestinal dysfunction, which are frequently reported among EBOV survivors. Although ocular involvement has been previously reported with both strains, the absence of auditory and enteric symptoms in our BVD survivor cohort calls for further comparative research on viral pathogenesis.

Immunologically, elevated basophil levels in 40 % of survivors, along with increased urinary leukocytes and ketones, suggest ongoing low-grade inflammation or innate immune reprogramming. These findings are consistent with previous insights indicating that survivors of filovirus infections may experience persistent immune activation even years after recovery [38]. Notably, monocyte and lymphocyte count normalised across both groups, and systemic inflammatory markers, such as CRP, were only mildly elevated. This reinforces the idea of subtle rather than overt immune dysregulation. While causality cannot be definitively assigned, these immune signatures highlight the necessity for deeper immunophenotyping, particularly of memory B-cell subsets, T-cell activation states, and exhaustion markers, given the known immunomodulatory effects of filoviruses during acute infection [18,39].

Despite these immune alterations, organ-specific laboratory markers, including hepatic enzymes (AST, ALT), renal indices (creatinine, urea), and electrolytes, demonstrated near-complete recovery. This contrasts with reports of prolonged hepatic and renal dysfunction in EVD survivors [40], again suggesting that BVD may induce a less severe post-infectious end-organ burden. However, the lack of consistent perturbations in blood glucose or HbA1c levels, with higher abnormalities observed among controls, was unexpected. These differences may be influenced by confounding lifestyle factors, including diet, physical activity, or healthcare access, rather than protective effects from BDBV exposure.

A novel physiological observation was the significantly lower respiratory rate among survivors, particularly in males, compared to matched controls. While values remained within normative ranges, this unexpected finding raises the possibility of long-term autonomic recalibration or altered respiratory drive post-infection. Such physiological adaptations have not been previously reported in BDBV or EBOV survivor cohorts and merit targeted cardiorespiratory evaluation in future studies. The absence of differences in cardiovascular indices (heart rate, blood pressure) or metabolic markers (BMI, temperature) supports overall homeostatic stability.

From a psychosocial perspective, our data reveal unexpectedly high mental health resilience: over 70 % of survivors scored within the normal range for anxiety, and over 60 % for depression, even after 16 years. This sharply contrasts with the substantial psychiatric morbidity, particularly PTSD and depression, reported in EVD survivor cohorts in Sierra Leone and the DRC [[41], [42], [43]]. Several contextual factors may explain this discrepancy: the smaller size and duration of the Bundibugyo outbreak, reduced media sensationalism, sustained community cohesion, and cultural resilience mechanisms like faith-based coping. The long-elapsed time since the outbreak may also have enabled psychological recovery that was not captured in shorter-term assessments of other outbreaks. Nonetheless, the persistence of stigma in 57.5 % of survivors indicates ongoing social exclusion, underscoring that mental health extends beyond symptom scores to encompass relational and societal domains.

Importantly, our study aligns with growing recognition that EBOD survivorship represents a chronic health state that requires tailored interventions across clinical, psychological, and immunological domains [10,14]. However, unlike the dominant focus on EVD, BVD survivors have remained underrepresented in post-viral care models and research pipelines. Our findings directly address this inequity and call for virus-specific survivorship guidelines, integrating longitudinal clinical monitoring, mental health support, and immunological surveillance.

Limitations include the cross-sectional design, which precludes assessment of symptom evolution and causal inference. Survivor-reported symptoms may be influenced by recall bias, and the sample size, though representative of the known BVD survivor population, is limited for high-resolution subgroup analyses. Residual confounding by socioeconomic factors, healthcare access, or undiagnosed comorbidities cannot be completely excluded. The absence of contemporaneous serological confirmation of control IgG status represents another limitation; however, given the 17-year interval since the outbreak and the expected waning of Ebola virus–specific IgG titres, serology would not reliably distinguish past exposure status. Classification based on verified Ministry of Health records and self-reported history, therefore, remained the most valid approach for this long-term survivor study. Future longitudinal investigations incorporating baseline comparisons and systems immunology approaches, such as single-cell transcriptomics, will be crucial for identifying biomarkers of resilience or pathology. Despite these limitations, the study offers critical and generalisable insights applicable to outbreak-prone, resource-limited settings. The integration of clinical, biochemical, immunological, and psychosocial dimensions positions this work to inform both survivor care policies and epidemic preparedness frameworks, particularly as the global health agenda shifts from reactive outbreak response to proactive, equity-focused pandemic resilience.

In conclusion, this study demonstrates that survivors of BVD experience persistent multisystem symptoms, subtle immunometabolic alterations, and psychosocial challenges more than a decade after infection. Despite clinical resolution of acute illness, chronic impairments, particularly in neurological, inflammatory, and social domains, persist, requiring deliberate integration into long-term care strategies. When interpreted alongside our previous work on SUDV and MARV survivors [11,12], the present findings reinforce the concept of “filovirus survivorship” as a chronic, multisystem health state transcending species boundary. These cross-cohort consistencies highlight the value of integrated longitudinal surveillance to inform virus-specific, yet harmonised, survivor-care and rehabilitation frameworks across the Filoviridae. From a policy standpoint, our findings validate BVD as a clinically significant post-viral condition, warranting inclusion in global therapeutic, diagnostic, and vaccine development agendas. Clinically, we advocate for the development of post-viral care models tailored to the unique features of BVD sequelae, models that integrate mental health, rehabilitative services, and longitudinal immune surveillance. Scientifically, the immune-metabolic recalibrations observed in our cohort warrant further exploration, potentially serving as a model for understanding chronic outcomes of other viral hemorrhagic fevers. As the global community pivots toward inclusive and forward-looking pandemic preparedness, our data reinforce the imperative for survivor-centered frameworks that not only contain viral spread but also uphold long-term human health and dignity.

## CRediT authorship contribution statement

**Raymond Ernest Kaweesa:** Writing – review & editing, Writing – original draft, Validation, Supervision, Project administration, Methodology, Investigation, Data curation, Conceptualization. **Joseph Ssebwana Katende:** Writing – review & editing, Validation, Supervision, Methodology, Investigation. **Raymond Reuel Wayesu:** Writing – review & editing, Writing – original draft, Visualization, Validation, Software, Formal analysis, Data curation. **Annie Daphine Ntabadde:** Writing – review & editing, Supervision, Project administration, Methodology, Investigation. **Solomon Opio:** Writing – review & editing, Validation, Project administration, Methodology, Investigation. **Laban Kato:** Writing – review & editing, Validation, Methodology, Investigation, Data curation. **Gerald Kevin Oluka:** Writing – review & editing, Validation, Project administration, Methodology, Investigation. **Ruth Nambi:** Writing – review & editing, Validation, Project administration, Methodology, Investigation. **Rodney Abraham Tumusiime:** Writing – review & editing, Validation, Project administration, Methodology, Investigation. **Pontiano Kaleebu:** Writing – review & editing, Validation, Supervision. **Julius Julian Lutwama:** Writing – review & editing, Validation, Project administration, Methodology, Investigation. **Jennifer Serwanga:** Writing – review & editing, Writing – original draft, Visualization, Validation, Supervision, Project administration, Methodology, Investigation, Funding acquisition, Formal analysis, Data curation, Conceptualization.

## Filovirus study team

Solomon Opio, Angella Namuyanja, Abel Ntale, Laban Kato.

## Ethical approval statement

This work was funded by the Coalition for Epidemic Preparedness Innovations (CEPI) under the Universal Protocol for Standardising Assays and Advancing Vaccine Immunogenicity Assessments for Emerging and Re-emerging Viral Threats, implemented through the Uganda Virus Research Institute (UVRI) as part of CEPI's Centralised Laboratory Network (CLN).

This research was partially financed by the Bill & Melinda Gates Foundation through the GIISER Uganda Grant (Investment ID INV-036306). The conclusions and findings given are the exclusive opinions of the writers and do not necessarily reflect the views or policies of the foundation.

## Data sharing

De-identified individual participant data that underlie the results reported in this article are available upon reasonable request to the corresponding author (Dr Jennifer Serwanga, Jennifer.Serwanga@mrcuganda.org). Access will be granted in accordance with the Uganda Virus Research Institute's (UVRI) data governance and ethical review procedures, contingent upon approval of a methodologically sound proposal. Data will be shared for purposes of academic, non-commercial research that aligns with the original study objectives and ethical commitments to participants.

## Funding source

This work was funded by the Coalition for Epidemic Preparedness Innovations (CEPI) under the Universal Protocol for Standardising Assays and Advancing Vaccine Immunogenicity Assessments for Emerging and Re-emerging Viral Threats, implemented through the Uganda Virus Research Institute (UVRI) as part of CEPI's Centralised Laboratory Network (CLN).

This research was partially financed by the 10.13039/100000865Bill & Melinda Gates Foundation through the GIISER Uganda Grant (Investment ID INV-036306). The conclusions and findings given are the exclusive opinions of the writers and do not necessarily reflect the views or policies of the foundation.

## Declaration of competing interest

The authors declare that they have no known competing financial interests or personal relationships that could have appeared to influence the work reported in this paper.

## Figures and Tables

**figs1:**
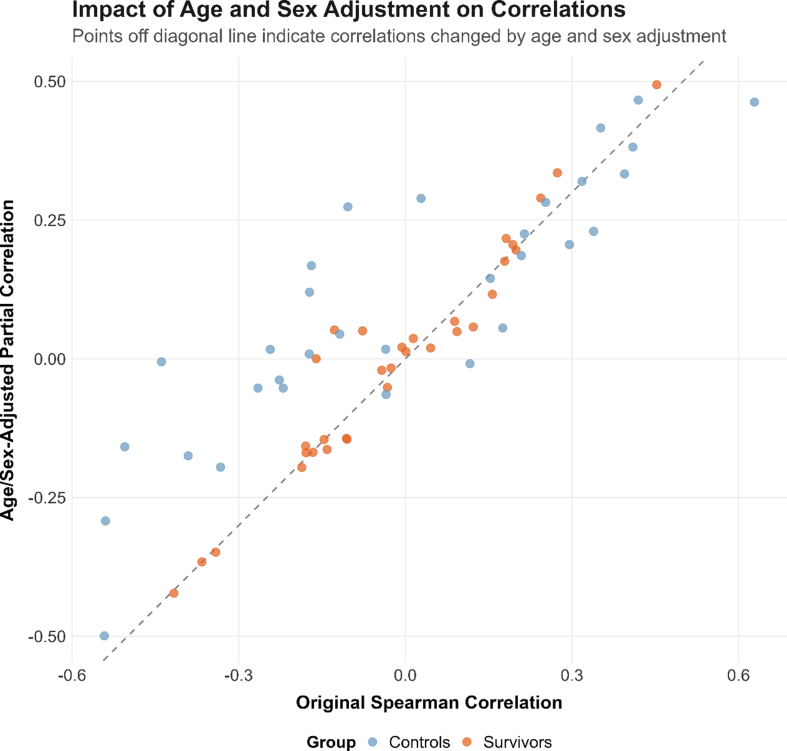


**figs2:**