# Supplementary material for: Resilience and residuals beyond containment — The hidden burden of Bundibugyo Ebola virus survivorship sixteen years on: A cross-sectional observational study
Source: New Microbes New Infect. 2025 Dec 13;69:101685. doi: 10.1016/j.nmni.2025.101685 (PMC13033831; doi:10.1016/j.nmni.2025.101685)
Supplement: Multimedia component 1 [file mmc1.docx]

STROBE Checklist, Cross-Sectional Study

Manuscript: Resilience and Residuals Beyond Containment — The Hidden Burden of Bundibugyo Ebola Virus Survivorship Sixteen Years On

| **Item No.** | **Recommendation** | **Section / Paragraph Where Addressed** | **Notes / Implementation in This Study** |
| --- | --- | --- | --- |
| Title and Abstract |  |  |  |
| 1 | Indicate the study’s design with a commonly used term in the title or the abstract. | Title, Abstract | Design stated as “cross-sectional observational study.” |
| Introduction |  |  |  |
| 2 | Explain the scientific background and rationale for the investigation. | Introduction, paragraphs 1–3 | Provides epidemiological/clinical rationale; highlights research gap in Bundibugyo Ebola virus disease (BVD) survivorship. |
| 3 | State specific objectives, including any prespecified hypotheses. | Final paragraph of Introduction | Objective: evaluate long-term clinical, biochemical, immunological, and psychosocial sequelae among BVD survivors vs unexposed controls. |
| Methods |  |  |  |
| 4 | Present key elements of study design early in the paper. | Study Design and Population | Described as cross-sectional observational design. |
| 5 | Describe the setting, locations, and relevant dates, including periods of recruitment and data collection. | Study Design and Population; Results: Study Population | Bundibugyo District, Uganda; recruitment Dec 2024–Feb 2025. |
| 6a | Give the eligibility criteria, and the sources and methods of selection of participants. | Participant Selection and Eligibility Criteria | Survivor and control inclusion/exclusion criteria based on Uganda MoH outbreak records and local documentation. |
| 6b | For matched studies, give matching criteria and numbers of exposed and unexposed. | Matching of Survivors and Controls | Frequency-matched by age group and sex; 40 survivors and 23 controls enrolled. |
| 7 | Clearly define all outcomes, exposures, predictors, potential confounders, and effect modifiers. | Data Collection and Clinical Assessment; Laboratory and Immunological Evaluation; Mental Health and Social Outcomes | Variables described; confounders (age, sex) adjusted in analysis. |
| 8 | Give sources of data and details of methods of assessment (measurement). | Data Collection and Clinical Assessment; Laboratory and Immunological Evaluation; Statistical Analysis | Instruments/assays and measurement standards specified (questionnaires, clinical assays). |
| 9 | Describe any efforts to address potential sources of bias. | Matching of Survivors and Controls; Limitations (Discussion) | Community-matched controls; demographic adjustment; addressed recall/selection bias. |
| 10 | Explain how the study size was arrived at. | Matching of Survivors and Controls, Demographic Balance, and Sample Size Considerations | Precision-based justification (Hedges & Olkin); effect size rationale (Supplementary Figure S2). |
| 11 | Explain how quantitative variables were handled in the analyses. | Statistical Analysis | Non-parametric tests; effect sizes (Hedges’ g); age/sex adjustments. |
| 12a | Describe all statistical methods, including those used to control for confounding. | Statistical Analysis | Wilcoxon, χ², Fisher’s exact, Spearman; partial correlations controlling for age/sex. |
| 12b | Describe any methods used to examine subgroups and interactions. | Statistical Analysis | Gender-stratified analyses; partial correlations. |
| 12c | Explain how missing data were addressed. | Statistical Analysis | Missingness <5%; available-case analysis; no imputation. |
| 12d | If applicable, describe analytical methods taking account of sampling strategy. | N/A | Census-like inclusion of all traceable survivors (n=40). |
| 12e | Describe any sensitivity analyses. | Statistical Analysis; Supplementary Figure S1 | Robustness via adjusted vs unadjusted correlations. |
| Results |  |  |  |
| 13a | Report numbers of individuals at each stage of study (e.g., eligible, examined, included). | Results: Study Population; Figure 1 | Numbers screened, excluded, and enrolled for survivors and controls. |
| 13b | Give reasons for non-participation at each stage. | Participant Selection | Two survivors deceased; seven controls excluded (age, refusal, incomplete data). |
| 13c | Consider use of a flow diagram. | Figure 1 | STROBE flow diagram provided. |
| 14a | Give characteristics of study participants and information on exposures and potential confounders. | Study Population and Demographic Characteristics; Figure 2 | Age, sex, occupation, marital status; group comparisons reported. |
| 14b | Indicate number of participants with missing data for each variable of interest. | Results: Mental Health | Missingness explicitly quantified (5%). |
| 15 | Report numbers of outcome events or summary measures. | Results; Figures 3–6 | Prevalence, severity, chronicity of symptoms; physiological and laboratory summaries. |
| 16a | Give unadjusted and confounder-adjusted estimates with precision (e.g., 95% CI). | Results; Supplementary S1 | Age/sex-adjusted partial correlations; effect sizes and p-values reported. |
| 16b | Report category boundaries when continuous variables were categorized. | Results (vital signs section) | Normal reference ranges specified. |
| 17 | Report other analyses done (e.g., subgroup, interaction, sensitivity). | Results; Supplementary Figures S1–S2 | Gender-stratified analyses; robustness checks. |
| Discussion |  |  |  |
| 18 | Summarize key results with reference to study objectives. | Opening of Discussion | Summarizes multisystem sequelae, immune-metabolic changes, psychosocial resilience. |
| 19 | Discuss limitations, considering potential bias or imprecision. | Limitations paragraph in Discussion | Addresses cross-sectional design, recall bias, small sample, socioeconomic confounders, IgG testing rationale. |
| 20 | Provide a cautious overall interpretation considering objectives, limitations, and other evidence. | Discussion (integrative paragraphs) | Compares with EBOV literature; highlights novelty and policy implications. |
| 21 | Discuss the generalisability of the study results. | Final paragraph of Discussion | Applicability to outbreak-prone, resource-limited settings; epidemic-preparedness frameworks. |
| Other Information |  |  |  |
| 22 | Give the source of funding and role of the funders. | Funding Source; Acknowledgements | CEPI and BMGF acknowledged; no role in design, analysis, or publication decision. |
| 23 | State ethical approval and consent procedures. | Study Design and Population; Ethical Approval Statement | UVRI-REC (GC/127/1045) and UNCST (HS5212ES); informed consent obtained. |
| 24 | Data availability and sharing statement. | Data Sharing | De-identified data available upon request, subject to UVRI data-governance approval. |
